# Psychological aspects of vestibular disorders: a national survey of clinical practice

**DOI:** 10.1017/S0022215124000458

**Published:** 2024-09

**Authors:** Laura J Smith, Wesley Pyke, David Wilkinson, Emma Travers-Hill, SS Surenthiran

**Affiliations:** 1 School of Psychology, Keynes College, University of Kent, Kent, CT2 7NP, UK; 2 Wolfson Institute of Population Health, Queen Mary University of London, London, EC1M 6BQ, UK; 3 The London Neuro-otology Centre, London, W1 G 6JL, UK

**Keywords:** cognition, mental health, outcome assessment, therapeutics, vestibular diseases

## Abstract

**Objective:**

This study examines how psychological aspects of vestibular disorders are currently addressed highlighting any national variation.

**Method:**

An online survey was completed by 101 UK healthcare professionals treating vestibular disorders. The survey covered service configurations, attitudes towards psychological aspects and current clinical practice.

**Results:**

Ninety-six per cent of respondents thought there was a psychological component to vestibular disorders. There was a discrepancy between perceived importance of addressing psychological aspects and low confidence to undertake this. Those with more experience felt more confident addressing psychological aspects. History taking and questionnaires containing one or two psychological items were the most common assessment approaches. Discussing symptoms and signposting were the most frequent management approaches. Qualitative responses highlighted the interdependence of psychological and vestibular disorders which require timely intervention. Barriers included limited referral pathways, resources and interdisciplinary expertise.

**Conclusion:**

Although psychological distress is frequently identified, suitable psychological treatment is not routinely offered in the UK.

## Introduction

The vestibular system comprises a complex set of inner-ear structures that detect the position and movement of the head to maintain spatial orientation and postural control. This information is integrated with visual and proprioceptive inputs to allow the eyes to fixate on a moving target (vestibulo-ocular reflex) and to stabilise posture during movement (vestibulospinal reflex).^1^ Disruption to the vestibular system can cause illusory sensations of motion (vertigo), unsteadiness, visual disturbances, hearing loss and nausea. Up to 60 per cent of those with a vestibular disorder also experience psychological distress.^2^ This encompasses cognitive problems affecting visuospatial abilities, memory and attention,^3^ and mental health disturbances such as anxiety, depression, agoraphobia and depersonalization.^4^

Multiple factors likely contribute to the onset of psychological distress in vestibular disorders. Vestibular signals project to multiple cortical and subcortical regions implicated in cognitive processing, autonomic function and emotion regulation, providing a neuroanatomical basis for the psychological distress reported by people with vestibular disorders.^5–7^ Psychological distress can also emerge as a secondary reaction to life-limiting vestibular symptoms, influencing behaviour (e.g. avoiding situations that evoke discomfort)^6^ and cognition (e.g. shifting attentional resources towards balance).^8^ There is also some evidence that patients with pre-existing cognitive or psychiatric conditions appear more vulnerable to vestibular conditions.^9^ Although the mechanisms remain unclear, what is apparent is that psychological aspects can adversely affect quality of life, daily activities and health outcomes.^10–14^

Despite the prevalence and effects of psychological distress, gaps remain in understanding and managing the psychological aspects of vestibular disorders. The National Institute for Health and Care Excellence (NICE) guidance acknowledges that for more complex dizzy patients, care should involve a referral to a multidisciplinary team (for example ENT consultant, physiotherapist, audiologist, counsellor, or psychologist).^15,16^ Further, The Department of Health suggest employing a psychiatrist and psychologist within such a service as part of best practice.^17^ Implementation of this guidance is complicated by patient volume and demography, clinician availability and wide variation in service provision across the UK.

The Department of Health guidance makes no specific recommendations for how to assess psychological aspects and no favoured model for formulating or managing psychological symptoms has been established. Therefore provision of psychological support is open to interpretation by individual healthcare professionals and organisations, which could contribute to variation in clinical practice.^10^

To improve psychological support for people with vestibular disorders, a first step is to gain better understanding of current practice within ‘usual care’. This will inform the development of best-practice guidelines, identify gaps in training provision required to effectively address psychological aspects, and provide a benchmark for evaluating new interventions tested within randomised controlled trials.^18^ Consequently, this study aims to determine current practices, attitudes and confidence in addressing the psychological aspects of vestibular disorders amongst healthcare professionals in the UK.

## Materials and methods

An online survey was iteratively developed with input from researchers, healthcare professionals and people with lived experience of vestibular disorders. The study received ethical approval from the Psychology Research Ethics Committee at the University of Kent (202116332980867285). Data were collected voluntarily with the option to remain anonymous.

### Participants and recruitment

Participants were practising healthcare professionals who provide clinical care for people with vestibular disorders in the UK. Participants were identified from the Ménière's Society Healthcare Professionals List^19^ which provides the names and contact details of professionals specialising in vestibular/ balance aspects. After removing duplicates and professionals outside the UK, 182 contacts remained. All 182 professionals received a description of and a link to the online survey for completion and to share with team members. Reminders were sent to those who did not respond within three weeks.

Special interest groups for allied healthcare professionals (The Association of Chartered Physiotherapists in Vestibular Rehabilitation, British Society of Neuro-Otology, The British Association of Audiovestibular Physicians, The British Society of Audiology, Vestibular Interdisciplinary Working Group) shared information about the study with their membership. Posts about the survey were also shared on social media and with our professional networks.

### Survey design

This cross-sectional survey utilised a semi-structured format comprising multiple-choice questions and free-text fields for elaboration. Jisc Online Surveys platform was used to capture the survey data. The survey launched on 5 October 2021 and closed 3 June 2022.

Survey items were informed by a previous survey framework by Klein et al.^20^ and designed in accordance with good practice guidelines.^21^ Patient and public involvement was embedded into the development of our research objectives and study design, and example survey content was discussed at a patient- and public-involvement meeting attended by seven people with vestibular disorders and their family members. After embedding patient- and public-involvement feedback, the authors drafted an initial set of survey items and shared these with health researchers (*n* = 2) and vestibular healthcare professionals (*n* = 2) from the authors’ networks who provided feedback on the relevance and number of items. The two healthcare professionals also piloted the survey commenting on clarity of expression, formatting and the ordering of items. A final version of the survey was then distributed.

The survey took approximately 20 minutes to complete. The full survey is available in the Supplementary Materials. The survey covered: (1) practice patterns including type of service, location of service, number of weekly appointments and professionals comprising the multi-disciplinary team; (2) respondent characteristics including professional background, training and their role within a service; (3) attitudes towards psychological aspects including perceived importance of psychological aspects within management of vestibular disorders and confidence to address these; (4) current clinical practice for cognitive and mental-health problems including identification, assessment, management, clinical pathways and referral methods; and (5) further comments.

### Analysis

Responses to closed questions were exported from Jisc Online Surveys into Excel and IBM SPSS, version 26, for analysis. Descriptive percentages and summary statistics were performed on responses to closed questions. Branching logic was utilised meaning the number of responses to some items varied. Results are presented as a percent of total answers. Exploratory chi-square tests and Spearman's rank correlations were conducted on nominal and ordinal data.

Written comments provided to five open-ended questions were exported to Excel, and then imported into NVivo qualitative data analysis software (QSR International Pty Ltd., Release 1.6.1, Doncaster, Australia). A qualitative analysis was performed using thematic analysis.^22,23^ Familiarisation with the dataset began by reading the responses and noting anything of potential interest in a shared document. These notes were used to develop a coding framework to describe the responses (see Supplementary Materials for coding framework). The coding framework was then uploaded to NVivo and applied to each written response by highlighting sections of text and assigning these a code. Two authors (LJS, WP) separately applied the coding framework to the dataset. Coding disparities were resolved by discussion with a third author (SSS).

## Results

### Practice patterns

**Figure 1.**
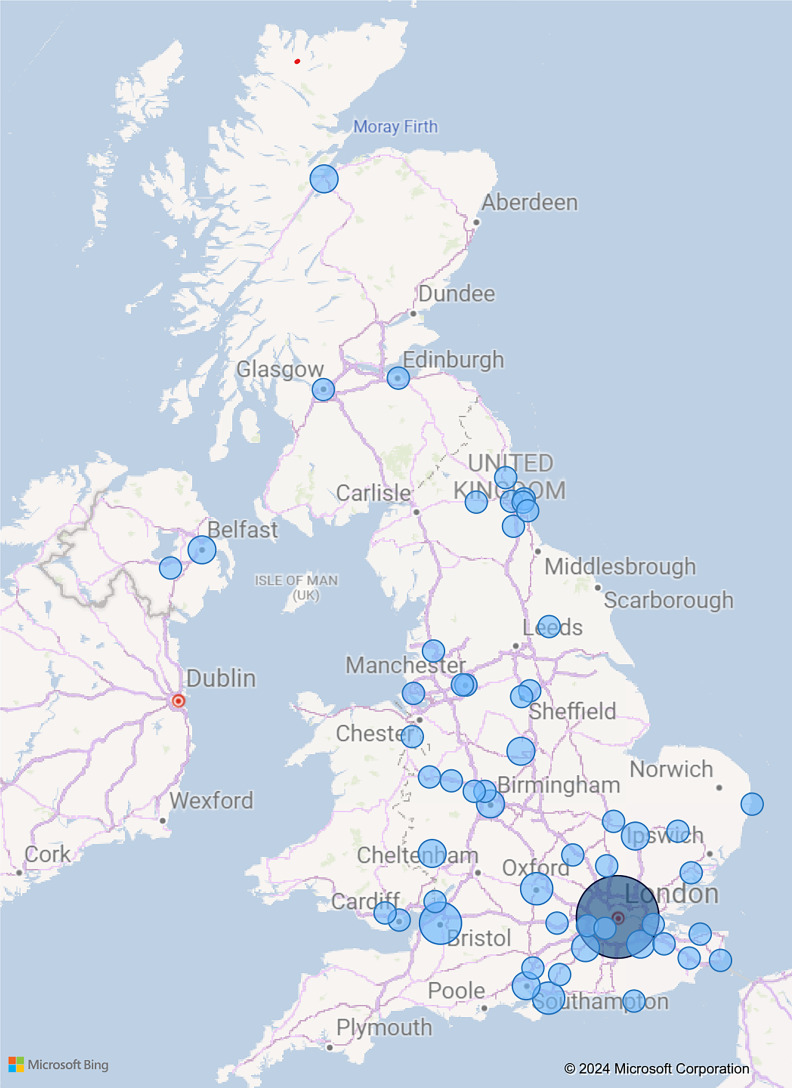
Responses received per geographical location across the UK. Larger bubbles represent a greater proportion of responses.

Responses were received from 103 participants. Of these, two were excluded because they were based outside the UK. Of the 101 respondents included in the analysis, 91 were based in England, 3 in Wales, 4 in Scotland and 3 in Northern Ireland. Figure 1 shows the geographical spread.

Most respondents practiced in a National Health Service (NHS) -funded service (*n* = 72). Other services were funded privately (*n* = 23), by charity organisations (*n* = 3) or other means (*n* = 3). One-third (*n* = 34) of respondents worked in a balance/ neuro-otology service, 22 in audiology, 13 in community/ outpatient neurorehabilitation, 11 in physiotherapy, 3 in falls, 3 in ENT, and 2 in general practicioner services. Thirteen participants fell into an “other” category. Most worked at the outpatient stage of the care pathway (*n* = 86), some also saw patients in a community setting (*n* = 30) or as inpatients (*n* = 17).

Some services comprised single disciplines (e.g., audiologist or physiotherapist), but most involved multi-disciplinary teams (e.g. audiologist, audio-vestibular physician, physiotherapist). On average services typically saw 63.13 (SD = 113.99, range 1–700) vestibular patients per week, suggesting both smaller (e.g. specialised tertiary) and larger (e.g. secondary care) services were captured.

### Respondent characteristics

Most respondents were physiotherapists, clinical scientists, and audiologists (see Table 1). On average healthcare professionals had worked clinically with vestibular disorders for 13.44 years (SD = 8.82, range 5 months to 54 years). Most treated people with vestibular disorders on a weekly (*n* = 48) or daily (*n* = 34) basis.

**Table 1.** Respondent characteristics

Role of Healthcare Professional	N	%	Years of Experience (Mean ± SD)*
Audiologist	10	9.9	13.5 ± 6.3
Audio-vestibular physician	7	6.9	23 ± 16.1
Clinical psychologist	3	3	13.5 ± 5.6
Clinical scientist/researcher	14	13.9	13.3 ± 7.2
Counsellor	1	1	[0.0]
ENT practitioner	2	2	18.5 ± 2.1
General practitioner	2	2	15.0 ± 7.1
Neurologist	5	5	19.8 ± 14.7
Neuro-otologist	6	5.9	20.6 ± 9.2
Occupational therapist	1	1	[10.0]
Optometrist	2	2	7.5 ± 2.1
Physiotherapist	43	42.6	9.9 ± 5.9
Psychiatrist	1	1	[8.0]
Other	4	4	21.5 ± 4.0
Total	101	100	

* M (mean) and SD (standard deviation) absent for roles which apply to one respondent only; value reported in [].

Nearly all respondents (92 per cent) had received some post-registration training. Respondents obtained this via in-house (internal expertise) training (*n* = 30), outsourced formal training (*n* = 41) and/ or conferences/ day courses (*n* = 59). This frequently included distinguishing vestibular symptoms from other causes of imbalance and identifying psychological aspects of vestibular disorders (see Supplementary Materials Table S1). Training on cognitive assessments and delivering therapies was uncommon.

### Attitudes towards psychological aspects

**Figure 2.**
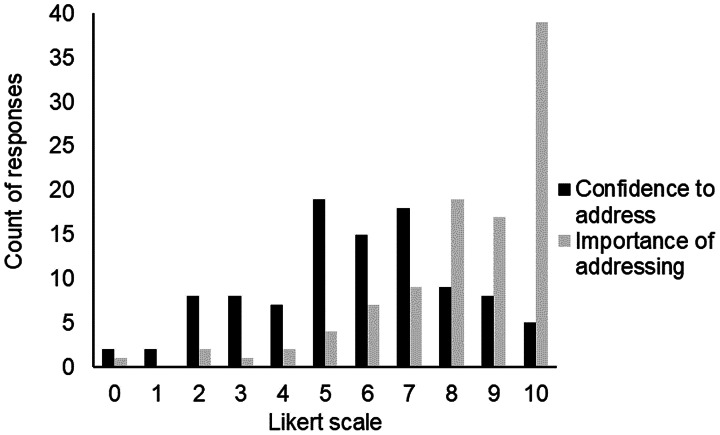
Perceived importance and confidence of addressing psychological aspects within care of people with vestibular disorders.

In response to the question “In your experience, do you think there is a psychological component to vestibular conditions?”, 97 respondents answered “yes”, the remaining 4 answered “not sure”. Participants also were asked to rate the importance of psychological aspects within vestibular care using a 10-point Likert scale ranging from “not at all important” to “very important”. Responses are plotted in Figure 2 (mean = 8.27, SD = 2.08), these were non-normally distributed with a negative skew (skewness = −1.58, kurtosis = 2.72). Perceived importance was neither associated with years of experience working with people with vestibular disorders (*r*(101) = −0.007, *p* = 0.94), nor with how frequently respondents work with vestibular disorders (*r*(101) = 0.092, *p* = 0.36).

Respondents were asked how confident they felt addressing psychological aspects within vestibular care using a 10-point Likert scale ranging from “not at all confident” to “very confident”. Responses are plotted in Figure 2 (mean = 5.69, SD = 2.38), the distribution was nearly normal (skewness = −0.25, kurtosis = −0.43). Confidence was positively associated with perceived importance (*r*(101) = 0.412, *p* < 0.001) and with years of experience working with vestibular disorders (*r*(101) = 0.277, *p* < 0.05). Confidence was not associated with how frequently respondents work with vestibular disorders (*r*(101) = −0.016, *p* = 0.873).

### Current clinical practice

#### Identification and assessment

Cognitive problems (*n* = 71) were identified or addressed less frequently than mental health problems (*n* = 80) [Χ^2^ (4, *n* = 101) = 13.051, *p*<.05]. Both were frequently addressed in the same service (*n* = 61). Some participants were unsure whether cognitive or mental-health problems were addressed in their service (both *n* = 12). Others answered ‘no’ and were not shown further questions about cognitive (*n* = 18) or mental-health (*n* = 9) problems.

Problems were mostly identified via self-report from the patient (cognitive *n* = 76; mental health *n* = 89) or family member (cognitive *n* = 65; mental health *n* = 73) or observed by the healthcare professionals (cognitive *n* = 69; mental health *n* = 84). Approximately one-third identified problems via routine screening/ assessment (cognition *n* = 18; mental health *n* = 35).

**Figure 3.**
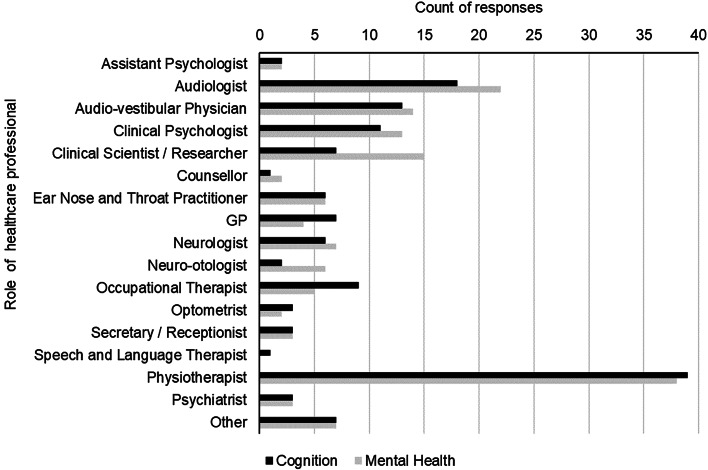
Role of healthcare professionals who identify and assess cognition and mental health in people with vestibular disorders; Multi-answer question, GP = General Practitioner

Cognitive and mental health problems were assessed by a range of healthcare professionals (not limited to psychology professionals; Figure 3). More than half of the respondents listed more than one professional who would assess cognition (*n* = 59) and mental health (*n* = 54).

Clinical interviews or history taking (*n* = 44) and questionnaires containing one or two items related to cognitive problems (*n* = 50) were the most common means of assessing cognition. Example reported questionnaires included the Dizziness Handicap Inventory, Vertigo Symptom Scale and Vestibular Rehabilitation Benefit Questionnaire. Fewer reported administering routine cognitive screening tools (*n* = 12), examples included the Montreal Cognitive Assessment and Mini–Mental State Examination. Detailed cognitive assessments were infrequently applied (*n* = 9), examples included the Wechsler Adult Intelligence Scale and Wechsler Memory Scale.

Questionnaires were often administered alongside comprehensive history taking to assess mental health. These encompassed questionnaires containing one or two items related to mental health (*n* = 64), examples included the Dizziness Handicap Inventory, Vestibular Rehabilitation Benefit Questionnaire, Situational Vertigo Questionnaire and The Nijmegen Questionnaire. Questionnaires focusing on mental health (*n* = 31) were also implemented including the Hospital Anxiety and Depression Scale, Beck Anxiety and Depression Inventories, Patient Health Questionnaire and General Anxiety Disorder Questionnaire.

### Management and intervention

Strategies used to manage psychological distress in people with vestibular disorders are shown in Table 2.

**Table 2.** Strategies used to manage cognitive and mental-health problems

Strategy	Cognition		Mental Health	
	N	%	N	%
Discuss symptoms with the individual	68	81.9	81	88
Report findings to other therapists	45	54.2	55	59.8
Give specific information/leaflets	22	26.5	41	44.6
Offer psychoeducation	13	15.7	15	16.3
Signpost to other relevant services & resources	63	75.9	69	75
Develop and implement a cognitive rehabilitation plan/psychological formulation	13	15.7	14	5.2
Adapt how other kinds of rehabilitation are delivered	43	51.8		
Offer compensatory cognitive strategies to support other rehabilitation	44	53		
Relaxation/mindfulness			54	58.7
Pharmacological approaches (prescribed)			19	20.7
Other	6	7.2	7	7.6
Not applicable	4	4.8	4	4.3

Multi-answer question; grey = N/A, question specific to cognition or mental health.

Discussing symptoms and signposting were the most frequent steps taken to address psychological aspects. Some respondents used resources from charity organisations (including Vestibular Disorders Association, Ménière's Society, Mind.org.uk/) or condition-specific websites (neurosymptoms.org, vestibularmigraine.co.uk) to provide information. Others utilised in-house resources such as leaflets on relaxation and lifestyle. Patients were also directed to other services (Improving Access to Psychological Therapies, Community Dementia, Mind) via leaflets and websites.

Some described specific interventions provided by their service including goal setting, pacing and coping strategies for cognition, along with Cognitive Behavioural Therapy, Acceptance and Commitment Therapy, mindfulness and relaxation to address mental health. Others did not provide specific detail but stated that such interventions would be tailored to individual need or depend on available provision.

### Clinical pathways and referral methods

Of the 83 respondents whose service identified or addressed cognition, 36 referred people with cognitive problems onto another practitioner. Of the 92 respondents whose service identified or addressed mental-health problems, 56 referred onto another practitioner. This referral was often made to a practitioner based in another service, which sometimes needed to go via the patient's general practitioner (see Table 3). There were no differences in referral methods between those working in private versus NHS services (cognitive: Χ^2^(6, *n* = 36) = 3.88, *p* = 0.69); mental health: (Χ^2^(9, *n* = 56) = 4.81, *p* = 0.85).

**Table 3.** Referral methods and role of practitioners within the referral pathway

	Cognition		Mental Health	
	N	%	N	%
Referral Method				
Specialist within the service	6	16.7	9	16.1
Specialist in another service	12	33.3	22	39.3
Recommendation to General Practitioner	6	16.7	18	32.1
A combination of the above	12	33.3	7	12.5
Total	36	100	56	100
Role				
Assistant Psychologist	1	2.8	3	5.4
Clinical Psychologist	17	47.2	21	37.5
Counsellor	4	11.1	11	19.6
Neuropsychologist	2	5.6	4	7.1
Occupational Therapist	3	8.3	0	0
Speech and Language Therapist	0	0	0	0
Psychiatrist	2	5.6	5	8.9
Other	7	19.4	12	21.4
Total	36	100	56	100

The roles of the practitioners to whom patients were referred are displayed in Table 3. Respondents who selected ‘other’ specified a hearing therapist, nurse practitioner, and Cognitive Behavioural Therapy counsellor. Some were unsure since this decision lay with the general practitioner. Referrals to this practitioner were infrequent, with most referring 5 or fewer patients per month (cognition *n* = 26; mental health *n* = 41), or 5 to 10 patients per month (cognition *n* = 6; mental health *n* = 7).

**Figure 4.**
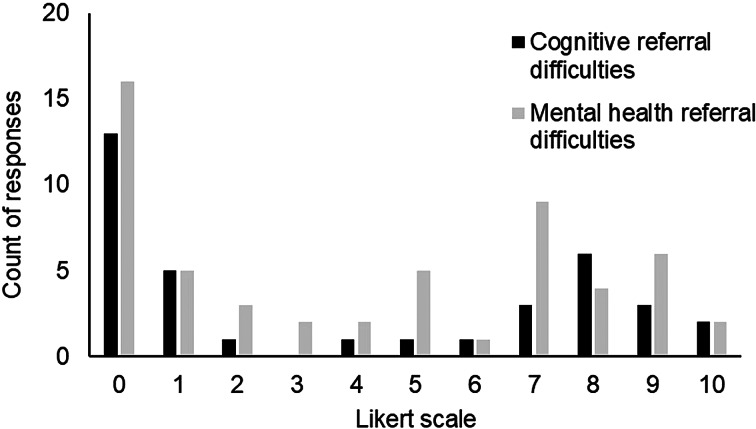
Perceived difficulty making referrals to a practitioner to address psychological aspects.

When asked how difficult it is to make a referral to this practitioner, using a 10-point Likert scale ranging from “not at all difficult” to “very difficult”, responses were variable (see Figure 4). Barriers to referrals included long waiting times, and the complexity and lack of clarity around referrals. The appropriateness and expertise of the person to whom the patient was referred were also highlighted (see Supplementary Materials Table S2).

### Qualitative analysis

Three themes were extracted from the 159 written comments that were provided: (1) interdependence of vestibular and mental health, (2) practitioner capacity and competency, and (3) pathways to treatment. Each theme and sub-theme are elaborated below with example quotes from respondents.

#### Interdependence of vestibular and mental health

This theme encompassed interactions between vestibular and mental health disturbances and the importance of understanding these connections. Mental health was thought to have a profound effect on the perceived burden of vestibular symptoms and ability to carry out daily activities. Respondents thought of mental health as a mediator which affected patients’ engagement with clinical treatment and could act as a potential barrier to rehabilitation.

“Things like anxiety often appear to underlie conditions/ symptoms or are exacerbated by them and I think are a really significant factor often influencing people's choice of coping strategies.” (audiologist, respondent_37)

“Levels of anxiety and panic disorder are high in this patient group, [which] influences engagement with rehab and recovery.” (physiotherapist, respondent_21)

Those who had experienced delays (e.g. long waits for appointments) or setbacks to treatment (e.g. incorrect diagnoses) were thought to experience mental distress more frequently and intensely.

“This long wait without treatment or correct management of their dizziness leads to poor (or non-existent) compensations strategies and increased anxiety and depression.” (clinical scientist/ researcher, respondent_27)

Conversely, respondents implied timely intervention could reduce mental health problems.

“If their symptoms are not addressed quickly, taken seriously, acknowledged as being disabling then psychological symptoms would certainly increase.” (audiologist, respondent_13)

Respondents described the importance of acknowledging and validating patients’ experiences. This included explaining that mental health problems are frequently experienced by people with vestibular disorders and the two often go “hand in hand” (counsellor, respondent_31).

“The approach I most commonly take is to ‘normalise’ their anxiety – reflecting that it is no surprise if the world turns upside down.” (neurologist, respondent_15)

Some respondents thought it was helpful to explain the connection between the vestibular and psychological systems, so patients understand how their biology and past experiences might influence their feelings and behaviour.

“Important that patients understand impact of anxiety/ fight and flight/ thought processes that can lead to increased symptoms.” (physiotherapist, respondent_89)

### Practitioner capacity and competency

This theme described factors influencing respondents’ ability to address psychological aspects within their role.

Respondents raised concerns around the complexity of psychological distress, recognising various psychosocial factors may interact to cause mental-health and cognitive disturbances. For example, previous trauma could have a lasting detrimental effect on mental health. Pre-existing and comorbid health conditions can also influence current clinical presentation.

“Patients want their problems to go away but may in some cases hold onto their problems as a mask for the psychological issues lying beneath. It seems to me that deep-rooted psychological problems do not go away.” (audiologist, respondent_42)

These complex interactions affected whether respondents felt able to help their patients. Understanding these complexities left some respondents feeling overwhelmed.

“When patients are very anxious, depressed and report multiple health issues I often am at a loss how to comprehend the mass of self-perceived problems and provide meaningful assistance.” (audiologist, respondent_42)

Respondents reflected on their professional boundaries including perceived responsibility and skillset for addressing psychological aspects.

“I have level 2 psychology training, but I feel that such disorders are so impactful that it often goes beyond my remit.” (specialist nurse, respondent_53)

Respondents also considered which activities they felt competent to deliver. Some felt confident to acknowledge and discuss psychological aspects but did not feel competent to treat these.

“I feel I can recognise disorders and distress, acknowledge their presence and relevance but then am limited in the support I can give with these issues.” (physiotherapist, respondent_69)

Some respondents had attended training and development events which equipped them with knowledge to address psychological aspects.

“There is a team within the trust which specialises in [Cognitive Behavioural Therapy] and mental health input …They have given us training on who to refer on and use of questionnaires.” (physiotherapist, respondent_60)

However, others wanted “more education/ resources” (physiotherapist, respondent_70) in this area to enhance their professional development.

“I have been doing this job a long time and I feel I am quite good at assessing psychological state without having had much training … It is an area that I would like to develop further.” (physiotherapist, respondent_95)

### Pathways to treatment

Pathways to treatment included factors that facilitated or hampered a patient's care journey. Respondents reflected on the capacity of their service to address psychological aspects given pressured workloads and limited appointment durations.

“When I was in the NHS it was much more difficult due the long waiting lists and shortage of practitioners and limited sessions.” (clinical scientist/ researcher, respondent_50)

Respondents faced practical challenges referring patients to appropriate psychological services. Few referral options were available, and these often had long wait times.

“Although recognised and sought routinely I have no clear pathway/ service to refer into for these patients.” (neuro-otologist, respondent_03)

“Very difficult to interest mental health services with these patients, especially since coronavirus disease 2019! Past attempts to involve clinical psychology services abandoned due in part to inability to persuade NHS managers to fund such services.” (audio-vestibular physician, respondent_04)

Available referral pathways were often indirect and lacked clear guidelines. For many, the only available pathway was via the general practitioner or self-referral to Improving Access to Psychological Therapies programmes. Both pathways were described as problematic in terms of actioning and monitoring the outcome of the referral.

“No confirmation that the general practitioner has acted on my recommendation.” (clinical scientist/ researcher, respondent_44)

“Wish we had in-house access to neuropsychology. I have to suggest referral to Improving Access to Psychological Therapies and that relies on patient making contact.” (physiotherapist, respondent_75)

Respondents thought having direct pathways to a psychology professional within the same service would improve care provision.

“We struggle to get access to the appropriate services for these patients that require it. No in-house NHS psychology for our balance patients.” (physiotherapist, respondent_66)

Being able to refer to healthcare professionals with specialist knowledge of connections between the vestibular and psychological systems was also highlighted.

“We need more psychologists with training in vestibular disorders to refer to.” (audiologist, respondent_39)

Respondents commented on this specialist knowledge being important to ensure patients engage and benefit from psychological therapies.

“Often services are generic … So many balance patients report that the services are not specific enough to help with their problem.” (neuro-otologist, respondent_29)

Some respondents felt referring to a non-specialist could hinder recovery if conflicting information is relayed causing confusion, or if patients feel misunderstood.

“I am often reliant on use of community mental health teams and Improving Access to Psychological Therapies services, which can be counterproductive at times due to their unfamiliarity with more complex variations/ influence of bi-directional impact.” (audio-vestibular physician, respondent_58)

## Discussion

To our knowledge, this is the first study to examine how psychological distress is currently addressed by UK healthcare professionals working with vestibular disorders. Most respondents thought there was a psychological component to vestibular disorders that was important to address, consistent with growing evidence of the effect of psychological distress in vestibular disorders.^24^ However, perceived confidence to address psychological aspects was variable and not rated particularly highly. The high importance ratings combined with responses in the ‘interdependence of vestibular and mental health’ theme indicate that this discrepancy was not due to lack of awareness or motivation among healthcare professionals.

Confidence was related to greater experience working with vestibular disorders; some respondents had less than one year of experience while others had more than 50 years of experience. Prior training is also likely a factor since few respondents had received post-registration training on psychological assessments and delivering therapy techniques. Additional training opportunities and mentoring from experienced colleagues may help to reduce this discrepancy by equipping staff with knowledge and skills needed to address psychological aspects and in turn enhance their self-efficacy.^25,26^

Lack of consensus surrounding the assessment of psychological aspects also could have influenced confidence and clinical practice. Respondents specified 20 different tools to capture cognition and 24 for mental health. This national variation makes it difficult to compare individuals and creates barriers for clinical decision making.^20^ The Dizziness Handicap Inventory was the most frequently used tool, perhaps because it is quick to complete and contains items related to functional and emotional handicap which are associated with anxiety and depression.^27^ However, other measures are available which specifically target cognition and mental health (e.g., Neuropsychological Vertigo Inventory, Patient Health Questionnaire). Importantly, spatial abilities (navigation, learning, recognition), which appear particularly sensitive to vestibular dysfunction, are absent from the Dizziness Handicap Inventory.^28^ Cut-off scores from the Dizziness Handicap Inventory (mild, moderate, severe) could be used to facilitate clinical judgements about when to conduct targeted assessments or refer for further specialised evaluations. Moving forward, a Delphi consensus study conducted with diverse stakeholders would help to develop a recommended set of assessments that can be routinely applied to screen for cognitive and mental-health problems.

Our findings indicate that the availability and lack of clarity surrounding referral pathways also influences respondents’ perceptions and practices relating to psychological aspects. Referral pathways varied depending on local provision and expertise but typically were to professionals within another service, often via the patient's general practitioner. Respondents felt access to integrated in-house psychological services would facilitate care provision and break down barriers associated with patients self-referring and relying on pressured general practitioners.^29^ This aligns with best practice recommendations from the Department of Health^17^ who advocate having a dedicated psychiatrist and a psychologist within specialist balance services. Our data show little evidence of this being implemented in clinical practice. Current pressures on UK healthcare services, including the growing demand for psychological services coupled with unfilled vacancies, mean this is unlikely to be implemented.^30,31^

Additional barriers included shortage of psychology professionals with relevant expertise of vestibular disorders, across both private and NHS services. Given the complex (two-way) interrelationship between vestibular and psychiatric conditions, psychological distress needs to be addressed within the context of the vestibular condition.^32^ Psychological interventions which apply standardised therapeutic protocols in isolation are therefore unlikely to be as effective or perceived as relevant by patients.^33,34^ This highlights the need for healthcare professionals to share their expertise of balance disorders within trainee curricula and post-qualification professional-development opportunities, and for further research to explore the active psychological mechanisms of change in vestibular disorders to enable targeted interventions to be developed.^35^

With regard to therapeutic strategies implemented, discussing symptoms and signposting were the most frequent steps taken to manage psychological aspects. Qualitative responses offered insights into the nature of those discussions which tended to (1) acknowledge psychological aspects, (2) explain how the vestibular and psychological systems interrelate, and (3) validate patients’ concerns with an empathetic response. Respondents also signposted to educational resources (leaflets, websites) to help patients become informed and learn coping strategies (e.g. relaxation/ mindfulness techniques). This aligns with previous research showing that psychoeducation can help prevent symptoms worsening by managing patients’ expectations and promoting engagement with treatment.^36^

Excellent resources are already freely available (e.g. from the Ménière's Society, Life on the Level, VEDA, vestibularmigraine.co.uk) to facilitate signposting, although our data indicate further dissemination is required to ensure these are utilised. Importantly, existing research indicates self-help approaches are optimal when delivered in collaboration with healthcare providers.^37^ Monitoring engagement with these self-help resources may help to maximise therapeutic benefit and fill resource gaps where psychological services are limited.

A strength of our study is the range of topics covered and the combination of quantitative and qualitative data, providing novel insights into clinical practice. However, the survey may be biased towards people with an interest in psychological aspects. To reduce bias, we employed several recruitment strategies including contacting individuals listed on the Ménière's Society Healthcare Professionals List and via mailing lists of special-interest groups. By offering participants the option to remain anonymous we hoped to reduce social desirability bias. However, this meant we were unable to calculate accurate response rates from particular recruitment strategies, such as the Ménière's Society Healthcare Professionals List.

Branching logic helped reduce demands on respondents, however this meant that some respondents did not see all the items. For example, those who did not address cognitive/ mental-health problems in their service were not asked about referral pathways and difficulties which may have led us to overlook important viewpoints. Our survey was designed to generate a relatively large dataset while limiting the time commitment of respondents. Corroborating data will help elucidate current clinical practice.

- Approximately 60 per cent of people with vestibular disorders are thought to experience psychological distress encompassing mental health disturbances and cognitive problems
- Although the need for psychological input is widely acknowledged, clinical guidelines are limited
- Healthcare professionals recognised the importance of addressing psychological aspects but lacked the confidence to undertake this
- Psychological distress was frequently identified by healthcare professionals, but psychological treatment was not routinely offered
- Training, experience, expertise and appropriate referral pathways affect assessment and management practices

There is growing appreciation of psychological distress in vestibular disorders, however there are no specific, practical or appropriate assessment and treatment guidelines in place, leading to national variation and unmet clinical needs. While, healthcare professionals recognised the importance of psychological aspects, and most could identify psychological distress, they lacked the confidence and expertise to manage psychological distress effectively and appropriately. Vestibular disorders are common and can have considerable consequences for the patient, their family unit and society (including unemployment and pressured healthcare services).^38^ Psychological distress not only compounds patients’ suffering, but also impedes treatment of the underlying vestibular disorder, prolonging recovery times and thereby adding more pressure on strained healthcare systems. Therefore, addressing the issues raised in this study by providing adequate training, effective referral pathways and appropriate service provision is a priority.

## Supporting information

Smith et al. supplementary material Smith et al. supplementary material

## Supplementary material

The supplementary material for this article can be found at https://doi.org/10.1017/S0022215124000458.
