# Supplementary material for: Psychological aspects of vestibular disorders: a national survey of clinical practice
Source: J Laryngol Otol. 2024 Apr 22;138(9):884–92. doi: 10.1017/S0022215124000458 (PMC11518673; doi:10.1017/S0022215124000458)
Supplement: Smith et al. supplementary material [file S0022215124000458sup001.docx]

**Supplementary Materials**

**Full Survey Questionnaire**

Link to full survey questionnaire: <https://osf.io/279ge/?view_only=38697967b3e0490eaeea91284a701ec5>

**Coding Framework**

| **Code** | **Description** |
| --- | --- |
| **Patient aspects** | |
| **Psychological aspects experienced** | |
| Anxiety | Worried, stressed, apprehensive, panic, avoidance, frightening, hyperventilation, PTSD, fear of falling. |
| Depression | Low mood, unhappiness, hopelessness, apathy. |
| Grief | Loss, grief at no longer being able to meet the expectations of self or others. |
| Frustration | Irritability, anger, annoyance. |
| Fatigue | Feeling mentally/ physically tired or drained. |
| Other |  |
| **Impact of psychological aspects** | |
| On vestibular symptoms | Hold onto vestibular symptoms to mask psychological, increase suffering, symptom monitoring. |
| On diagnosis | Autonomic dizziness symptoms being mistaken for anxiety, unclear whether presenting with vestibular or anxiety response. |
| On rehabilitation and recovery | Reduce engagement, negative outlook, order in which vestibular and psychological aspects are addressed. |
| Broader impacts | Functioning, quality of life, activities of daily living, acceptance, disabling, work, relationships, social isolation. |
| Other |  |
| **Individual differences** | |
| Chronicity | Time taken to diagnose, what starts as a vestibular insult/ disorder progresses to psychological distress over time, chronic problems lingering between attacks. |
| Psychological factors | Help seeking behaviours, coping, illness perceptions. |
| Severity of psychological aspects | Not all patients are affected, spectrum of psychological aspects, mild/ moderate/ severely affected. |
| Other health concerns | Concurrent health conditions, longstanding mental health conditions, previous trauma, multimorbidity. |
| Other |  |
| **Complex interplay of vestibular and psychological systems** | |
| Distressing vestibular symptoms | Sudden onset, fear inducing, monitoring, anticipation, embarrassment. |
| Causal mechanisms | Are psychological aspects a primary cause of vestibular disorders or a secondary complication? What is the underlying association? |
| Interconnected systems | Psychological aspects can trigger/ exacerbate vestibular symptoms, shared neural networks, interoception, holistic approach. |
| Other |  |

| **Code** | **Description** |
| --- | --- |
| **Clinical practice** | |
| **Patient-clinician relationship** | |
| Communication | Active listening, taking time to talk with patients, accurately interpreting patients’ comments. |
| Trust | Rapport, honesty, building a therapeutic alliance. |
| Validation | Acknowledge, normalise, reassure, understanding, empathy. |
| Other |  |
| **Clinicians’ confidence relating to psychological aspects** | |
| Activities within professional remit or they feel comfortable undertaking | Recognising and discussing psychological impacts, validating and normalising experiences, using questionnaires, offering general advice. |
| Activities outside professional remit or they feel unequipped for | Severe or longstanding mental illness, where to refer patients, treatment and management of psychological aspects, self-harm. |
| Other |  |
| **Professional development** | |
| Training previously received | Psychological aspects of tinnitus, psychological questionnaires, basic mindfulness. |
| Training needs | Awareness of psychological aspects, assessment of psychological state, how to support, therapeutic techniques and resources. |
| Who would benefit from training | Vestibular healthcare professionals, mental healthcare professionals, primary care professionals. |
| How could training be delivered | Training courses, printed information, clinical supervision, joint clinics, MDT working, sharing expertise. |
| What would be the impact | Evaluating impact, outcomes, monitoring. |
| Other |  |
| **Resources** | |
| Appointments | Limited duration, limited number of sessions, capacity to assess and follow-up. |
| Long waiting lists | NHS staffing crisis, delays to treatment, delays lead to poorer outcomes. |
| Commissioning | No funding for psychology services for vestibular disorders, evidencing clinical need. |
| Private sector | Cost to patient, expensive, insurance, more direct routes to care. |
| Other |  |
| **Barriers to referral** | |
| Availability of appropriate psychology services | No service to refer into, local provision varies, no tailored resources, mental health services too generic and can be counterproductive, group/ online services may be unsuitable. |
| Availability of appropriate psychology healthcare professionals | Shortage of psychologists, no psychologists with specialist vestibular knowledge, expert management. |
| Cross-referral | Preference for in-house psychology, ability to treat within the team, dual approach, no pathways available. |
| Reliance on GP | Pressured workloads, awareness of psychological aspects of vestibular disorders, unclear whether GP has actioned requests. |
| Reliance on patient self-referral | Motivation, acceptance, illness burden. |
| Other |  |

Table S1. Relevant post-registration training received

| **Training Received** | **N*** | **%** |
| --- | --- | --- |
| Distinguishing vestibular disorders from other sources of imbalance | 83 | 82.2 |
| Having conversations about mental health and/or cognitive problems | 61 | 60.4 |
| Identifying mental health and cognitive problems | 36 | 35.6 |
| Administering and interpreting mental health interviews/ questionnaires | 33 | 32.7 |
| Administering and interpreting cognitive screening tests/ neuropsychological assessments | 14 | 13.9 |
| Delivering psychological therapy techniques | 22 | 21.8 |
| Delivering cognitive rehabilitation techniques | 24 | 23.8 |
| Other | 5 | 5 |
| No training received | 8 | 7.9 |

* Multi-answer question.

Table S2. Difficulties with referrals for cognitive and mental health problems

| **Referral Difficulties** | **Cognition** | | **Mental Health** | | |
| --- | --- | --- | --- | --- | --- |
|  | **N** | **%** | | **N** | **%** |
| Long waiting times | 20 | 55.6 | | 37 | 66.1 |
| Communication difficulties | 3 | 8.3 | | 7 | 12.5 |
| Complex referral procedures | 8 | 22.2 | | 14 | 25 |
| Lack of clear protocols for referrals | 9 | 25 | | 15 | 26.8 |
| Appropriateness of the person referring to | 6 | 16.7 | | 18 | 32.1 |
| Expertise of the person referring to | 7 | 19.4 | | 15 | 26.8 |
| Other | 4 | 11.1 | | 10 | 17.9 |
| No difficulties | 11 | 30.6 | | 9 | 16.1 |

* Multi-answer question.
